# Cardiopulmonary determinants of reduced exercise tolerance in Fabry disease

**DOI:** 10.3389/fcvm.2024.1396996

**Published:** 2024-05-02

**Authors:** Oriana De Marco, Jessica Gambardella, Antonio Bianco, Antonella Fiordelisi, Federica Andrea Cerasuolo, Antonietta Buonaiuto, Roberta Avvisato, Ivana Capuano, Maria Amicone, Teodolinda Di Risi, Eleonora Riccio, Letizia Spinelli, Antonio Pisani, Guido Iaccarino, Daniela Sorriento

**Affiliations:** ^1^ Department of Public Health, Federico II University, Naples, Italy; ^2^ Centro Interdipartimentale di Ricerca in Ipertensione Arteriosa e Patologie Associate, Federico II University of Naples, Naples, Italy; ^3^ Department of Advanced Biomedical Sciences, Federico II University, Naples, Italy; ^4^ CEINGE - Biotecnologie Avanzate, Naples, Italy; ^5^ Department of Clinical Medicine and Surgery, Federico II University, Naples, Italy

**Keywords:** Fabry disease, fatigue, exercise intolerance, cardiac dysfunction, pulmonary dysfunction

## Abstract

Fabry disease (FD), also known as Anderson-Fabry disease, is a hereditary disorder of glycosphingolipid metabolism, caused by a deficiency of the lysosomal alpha-galactosidase A enzyme. This causes a progressive accumulation of glycosphingolipids in tissues and organs which represents the main pathogenetic mechanism of FD. The disease is progressive and multisystemic and is characterized by early symptoms and late complications (renal, cardiac and neurological dysfunction). Fatigue and exercise intolerance are early common symptoms in FD patients but the specific causes are still to be defined. In this narrative review, we deal with the contribution of cardiac and pulmonary dysfunctions in determining fatigue and exercise intolerance in FD patients.

## 1 Introduction

Fabry disease (FD) is a rare and progressive genetic disorder characterized by the deficiency of α-galactosidase A enzyme, leading to the accumulation of glycosphingolipids in various tissues and organs. This multisystemic condition causes symptoms in many districts, including the skin, kidney, heart, and neurological system (1–5).

FD patients frequently experience fatigue, and numerous studies demonstrate that FD subjects have lower exercise tolerance than healthy controls, especially patients with peripheral neurological abnormalities (6). However, the precise causes of fatigue in FD and how it affects the effectiveness of exercise are still unknown (6).

**Figure 1:**
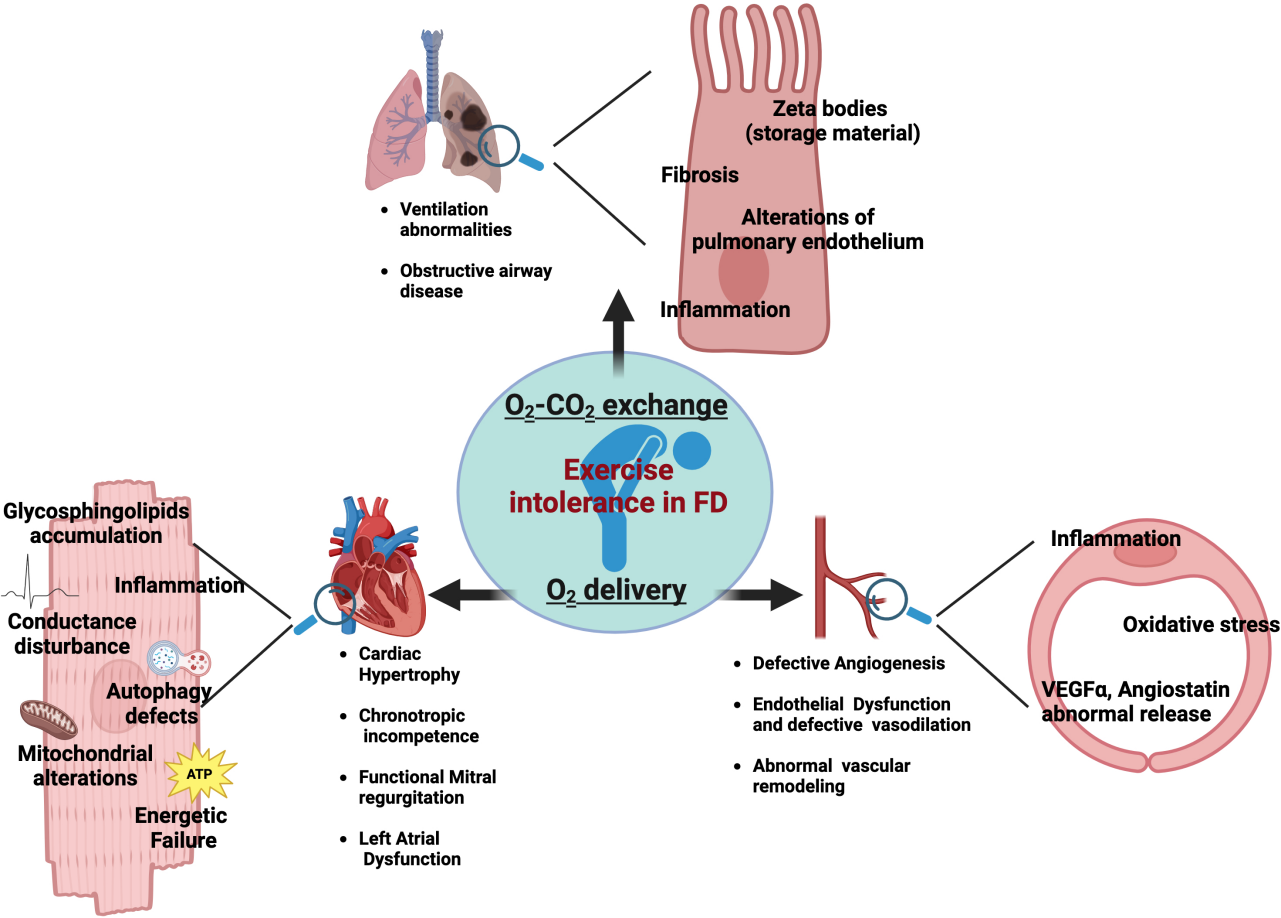
Main cardiopulmunary determinants of exercise intolerance in Fabry patients.

Fatigue is a complex phenomenon resulting from internal homeostasis breakdown in response to increased energetic demand by external stimuli. The mechanisms of fatigue are multifactorial as it can be influenced by a great variety of aspects. Some of these factors are modifiable, including lifestyle, while others are nonmodifiable, such as genetics, and sex. Indeed, fatigue susceptibility impacts men and women differently (7). In FD population, a wide spectrum of factors could synergically contribute to chronic fatigue. Primary muscle and metabolic alterations play an essential role in Fabry-related fatigue, alongside cardiac and respiratory dysfunctions which also have a strong impact on exercise tolerance and fatigability of FD patients. The detrimental impact of chronic fatigue on patients' quality of life highlights the need for further research to understand the underlying mechanisms and their synergic effects. In this issue, we specifically focus on the contribution of cardiac and pulmonary abnormalities in determining fatigue and exercise intolerance in FD patients (Figure 1). By a critical revision of the literature, we provide useful insights to diagnose and promptly manage fatigue in FD patients.

## 2 Exercise intolerance and fatigability in FD patients

An adequate exercise capacity is a complex response that requires the optimal interaction among heart, lung, vasculature, and skeletal muscle. Specifically, during physical effort the orchestration of the following processes is needed: (i) an adequate exchange of O_2_ and CO_2_ through pulmonary ventilation; (ii) an optimal function of the heart and vascular system to supply oxygenated blood at a sufficient flow rate to meet the metabolic demands of working muscles; (iii) efficient O2 diffusion, nutrients extraction and utilization in skeletal muscle (8). Alterations in any of these critical steps contribute to exercise intolerance and susceptibility to fatigue.

Fatigue and pain especially during effort is an emerging hallmark of FD (9). Commonly, this phenotype has an early onset and can precede FD-related organ damage (kidney, heart, lung) suggesting primary abnormalities of the motor system. However, the cardiovascular and pulmonary alterations also can contribute to exercise intolerance and fatigability in FD, especially in the late stage of the disease requiring specific prevention, management, and treatments. Indeed, although the exact underlying mechanisms are still unknown, it is certain that the reduced exercise capacity in FD is multifactorial and the specific vascular, cardiac and lung alterations described in FD patients could be potential contributors.

In the sections below we annotated the main cardiac and pulmonary morpho-functional alterations which can potentially contribute to exercise capacity limitation in FD.

### 2.1 Vascular determinants

Peripheral vascular function, especially endothelial homeostasis, is a key determinant of O2 delivery to muscle during exercise. Indeed, the impairment of endothelial dependent-vasodilation in response to aging and chronic illness is responsible for a reduction in systemic O2 delivery aggravating fatigue and dyspnea (10). Vascular tree alterations have been extensively described in FD patients. Specifically, endothelial and smooth muscle cells are among the main target of Gb3 storage (11), and drive the vascular manifestations in FD patients, including the basilar artery remodeling, increased intima-media thickness and decreased brachial flow–mediated dilation (12, 13). The vascular dysfunction strongly contributes to organ damage in FD exasperating kidney disease, cardiomyopathy, cerebral lesions and likely exercise capacity of skeletal muscle (9, 12, 14, 15). Systemic inflammation, oxidative stress and the abnormal release of angiogenic factors, including VEGFα and angiostatin, are considered the underling mechanisms of endothelial dysfunction in FD (16–18). Data about a direct correlation among endothelial function and exercise tolerance in FD patients are missing. However, already in the early stage of the disease, histological examination shows a significant presence in muscle vessels of glycosphingolipid accumulation (19). On this ground, we cannot exclude that the altered vessel homeostasis could affect O2 delivery, exacerbating muscle incompliance to physical effort. In this scenario, the well-established beneficial effects of physical activity on vessel homeostasis (20) further support the potential therapeutic power of an adapted physical activity program for FD patients.

### 2.2 Cardiac determinants

In health subjects, the increase of venous return during exercise is matched by an increased cardiac output through elevation in HR, contractility, and lusitropy and not in cardiac filling pressures (21). Specifically, the increased contractility in combination with vasodilation determines an enhanced end-diastolic volume and reduced end-systolic volume guaranteeing the match between systemic perfusion and muscle metabolic needs (8). An insufficient increase in cardiac output during effort leads to lactic acidosis and muscular fatigue limiting exercise and functional capacity (21, 22). In FD patients, cardiac alterations are not the exclusive but key determinants of exercise intolerance (8). More than 50% of FD patients show cardiac involvement including left ventricular hypertrophy, heart failure with preserved ejection fraction, chest pain, and arrhythmias (23, 24). FD register shows that cardiac damage is the first cause of death in FD patients and despite the late clinical onset, cardiac alterations start early in the life and progress sub-clinically (25). The buildup of glycosphingolipids and Gb3 occurs in all cardiac cell types including myocytes, endothelial and smooth muscle cells of intramyocardial vessels, endocardium, valvular fibroblasts, and conduction tissue probably culminating in inflammation, necrosis, fibrosis, and hypertrophic myocardial disarray (26, 27). However, Gb3 accumulation in the heart is *per se* not sufficient to explain the whole spectrum of cardiac manifestations and it has emerging the hypothesis that the primary enzymatic defect in FD triggers other processes that result in biochemical and functional alterations including autophagy alterations, mitochondrial defects, and energetic failure (25, 28). The energy depletion may activate pro-hypertrophic pathways, common to other hypertrophic cardiomyopathies, and may affect cardiac responsiveness to stress. Accordingly, the increase of energetic demand during physical effort represents a stress condition revealing the unsuitable energetic metabolism of the FD heart.

Chronotropic incompetence is another cardiac symptom frequently recorded in the FD population that could contribute to the exercise intolerance of FD patients. In healthy humans, during aerobic exercise, the VO_2_ increases approximately 4-fold, mainly through a significant increase in heart rate (2.2-fold) (29). Chronotropic incompetence, broadly defined as the inability of the heart to increase its rate in response to increased demand, is therefore, among the primary contributors of exercise intolerance (30). Approximately, 18%–20% of FD patients show chronotropic incompetence and/or sinus node dysfunction or severe atrioventricular block (31). In a cohort of 38 Australian patients with FD, 70% of subjects had resting bradycardia, with impaired ability to increase heart rate during exercise (32). Electrical alterations and conduction impairment could be involved in this phenomenon. Specifically, it has been proposed that glycosphingolipids accumulation may alter ion channel expression and/or cell membrane trafficking, affecting the electrical properties of cardiac cells (33). It should not be excluded that also energetic depletion can affect the functionality of ATP-dependent pumps, ionic homeostasis maintenance, and conductance capacity (34).

Functional mitral regurgitation as well is a key determinant of exercise intolerance in the general population. Despite the valve abnormalities are not the major limitations for cardiac function in the FD population, mild left ventricular valve regurgitations are commonly reported in FD patients (35). Indeed, the postmortem examination of Fabry hearts revealed that the greatest concentrations of glycosphingolipids were in the mitral valve (36). The most recent study revealed that in classic- FD, the prevalence of valvular disease, from moderate to severe, was 10%, with mitral and tricuspid regurgitation being the most common (37, 38). Beyond the glycosphingolipids accumulation specifically at valvular levels, other phenomenon could be involved in FD valve dysfunction, including thickening of the sub-valvular apparatus (35) geometric distortion of the atria, valvular annulus, or aortic root dilatation (37).

Emerging evidence implicates left atrial dysfunction as an important pathophysiologic mechanism of exercise intolerance (39). Specifically, it has been reported that LA stiffness is independently associated with impaired exercise tolerance and quality of life and may be an important therapeutic target in patients with heart failure with preserved ejection fraction. In FD population, studies of speckle-tracking echocardiography reveal that the left atrial reservoir, conduit, and contractile functions are all affected (40). Also, cardiac MRI studies show an impairment of left atrial function and morphological parameters already in the early stage of the disease (41), when effort tolerance is affected as well.

### 2.3 Lung determinants

While the main determinant in exercise tolerance is considered the heart, it is essential to recognize that limitation in lung function also contributes to the overall exercise capacity. Aerobic exercise increases oxygen and ventilation demands, leading to rapid and deep breathing, which can cause airway smooth muscle stretch, bronchodilation, and airway caliber maintenance (42). The multiorgan compromission in FD also includes the lung. Specifically, the pulmonary involvement in FD emerges as alterations of functional parameters, including the increase in resting dead space or ventilation abnormalities (43). Symptoms like coughing, wheezing, and shortness of breath are commonly reported in FD population, however, they could be also influenced by external factors such as smoking habits and age (44–46). Obstructive airway disease has been observed in a range of 27%–36% of FD patients across various cohorts, which is a higher prevalence compared to the general population (47).

The underlying mechanisms responsible for pulmonary function decrease in FD are still not fully elucidated. One hypothesis suggests that the accumulation of sphingolipids in the lung tissue, which in turn triggers an inflammatory response, may be responsible for mechanical damage and small airway disease (48). Rather, recent evidence suggests that pulmonary involvement in FD is indirect, linked to the lipid deposits occurring in vascular endothelium and bronchial smooth muscle, with subsequent obstruction of small airways. Also, in this case, the inflammatory process triggered by sphingolipids could serve as a crucial mechanism for mediating obstructive events (49). Even, Svensson et al. posited that the glycosphingolipids accumulation could activate a maladaptive remodeling of the bronchial tree with interstitial fibrosis and chronic airway limitation (27). Specifically, the obstructive events observed in FD patients could stem from either airway constriction due to smooth muscle hyperplasia or the accumulation of glycosphingolipids directly within bronchial cells (50, 51).

The electron microscopy analysis of sputum and lung biopsy samples from FD patients, revealed the presence of “myeloid-like” inclusions within ciliated cells (52). Additionally, lamellar inclusion bodies known as “Zebra bodies” were detected within the cytoplasm of ciliated bronchial epithelial cells (53). These inclusions were also found in bronchiolar/arteriolar smooth muscle cells and endothelium (54).

Overall, the pulmonary involvement in FD includes micro and macro-structural alterations producing a complex dysfunctional phenotype which still needs further research to be better characterized, as well as, the precise mechanisms responsible for lung function decline need to be delineated. Moreover, poor data are available on the effects of therapies on lung phenotype. Such studies including the report by Brier et al., showed an improvement in pulmonary function with ERT treatment (55), and the same results have also been shown in case reports with more critical situations (56).

### 2.4 The use of CPET to assess cardiopulmonary involvement in FD

The assessment of exercise tolerance is crucial to establish the overall health and fitness level of a subject. Cardiopulmonary exercise testing (CPET) is a comprehensive diagnostic test used to assess the integrated function of cardiovascular and respiratory systems during exercise. It involves incremental exercise, typically on a treadmill or stationary bike, while continuously monitoring various physiological parameters. These parameters include oxygen consumption, heart rate, blood pressure, ventilation, gas exchange (oxygen and carbon dioxide levels), and other relevant data (57–60). By measuring and integrating these data at different levels of physical effort, CPET provides valuable insights into an individual's exercise capacity, cardiorespiratory fitness, and any abnormalities or limitations in the cardiopulmonary system (61–64). Specifically, CPET provides parameters like heart rate, blood pressure, and cardiac output, allowing to identify alterations in anaerobic threshold and cardiac limitations. Contextually, Pulmonary limitations are detected through the analysis of oxygen uptake, ventilation, and gas exchange parameters, unveiling conditions like COPD or interstitial lung disease. Muscular fatigue could also be assessed by monitoring exercise capacity and an early onset of anaerobic metabolism. Overall, The CPET provides joint data analysis that allows complete assessment of the cardiovascular, respiratory, muscular and metabolic systems during exertion (57).

Therefore, the CPET is a useful tool to assess the impairment of cardiopulmonary homeostasis and function in FD patients. Specifically, the first consideration is that reduced exercise tolerance and fatigue are not only detectable in male patients where the clinical signs of FD are obvious (renal and cardiac dysfunction) but also in heterozygous female patients. Hence, CPET could unveil early and preclinical signs of cardiopulmonary dysfunction. For instance, Wang et al. investigated women with FD and recorded a reduced quality of life alongside fatigue in 58.5% and exercise intolerance in 82.5% of the participants. From this study, it emerges that a decrease in diastolic blood pressure greater than or equal to 10 mmHg was associated with exercise intolerance, while reduced maximal oxygen consumption correlated with fatigue. Moreover, during the stress test, the exercise intolerance reflected the decrease in maximal heart rate (65). A similar phenomenon was also described by Bierer et al. Their research revealed that approximately 46% of individuals with FD experienced a notable decline in diastolic blood pressure during exercise, especially among female patients (66).

To understand the relative involvement of heart and lung in reduced exercise tolerance, Spinelli et al. examined 16 patients with FD compared to control subjects, performing a radionuclide myocardial perfusion at rest and during exercise, tissue Doppler echocardiography, and magnetic resonance imaging (MRI) at rest. The participants were divided into two groups, according to their left ventricular mass and renal function parameters. The study revealed that patients with more severe organ damage exhibited abnormal stroke volume response to exercise, characterized by decreased end-diastolic volume and not reduced end-systolic volume. Moreover, compared with controls, FD patients had elevated plasma levels of NT-proBNP (a marker of cardiac stress), higher indexed left ventricular mass (LVMi), and altered parameters at tissue Doppler echocardiography, suggesting an advanced diastolic dysfunction. Overall, the study indicated that left ventricular hypertrophy and interstitial fibrosis play a significant role in affecting stroke volume response during exercise in FD, highlighting the impact of cardiac determinants in exercise intolerance of FD population (67).

The incompetence of the heart in supporting exercise-induced stress also emerged from the study by Réant and colleagues. The authors showed that FD subjects reach a lower mean peak oxygen consumption (VO_2_) and a higher VE/VCO_2_ slope compared to the general population, again confirming the reduction in cardiac output at peak exercise (68). Accordingly, Powell et al., by using CPET, showed a lower increase in heart rate at peak exercise in FD patients, alongside reduced indexed maximum oxygen consumption and indexed oxygen peak, with both maximal and submaximal testing criteria. The Authors also suggest the involvement of pulmonary circulation since a positive correlation between functional capacity and right ventricular volumes at cMRI was found. Indeed, although static imaging revealed normal systolic function, the impaired right ventricular stroke volume seemed to affect oxygen consumption (VO_2_) during exercise (69).

Overall, these findings indicate that CPET is a valuable tool to unveil cardiopulmonary alterations in FD even in the early stage of the disease, when the damage to cardiopulmonary systems is not yet full-blown or bland, for instance in women. The main results of all the studies evaluating exercise tolerance in Fabry patients are summarized in Table 1.

**Table 1:** Alterations of exercise performance parameters in FD patients and impact of interventions including ERT and exercise prescription.

	Parameters of exercise performance		
	Test performed	Untreated patients	Patients post- intervention
Bierer et al. (55)	CPET	Mean VO2max was 1.462 ± 0.25 L/min and decreased by 0.116 ± 0.44 L/min in untreated patients	In response to ERT: - Mean VO2max increased by 0.459 ± 0.64 L/min - Mean oxygen pulse (VO2/HR) increased by 1.71 -Estimated stroke volume (SV) increased by 10 ml
Lobo et al. (32)	Bicycle stress tests with VO(2) max measurement and once-only 6 min’ walk tests	Exercise capacity was reduced in FD compared with that predicted from normative population data.	In response to ERT: - Improvement of Anaerobic threshold - No changes in V02 max (different M/F, organ damage, genetic variants)
Tuan et al. (72)	Symptom-limited cycle ergometry	Peak exercise capacity of FD patients was lower than healthy controls. Peak of metabolic equivalent and of oxygen consumption decreased significantly over a period of 3 years in FD patients with cardiac variant	In response to ERT: - Stabilization of exercise capacity in patients with classic variant - No difference in patients with cardiac variant
Schmitz et al. (78)	Cycloergometry (stationary cycling) and isokinetics (resistance exercise).	Lower relative maximum performance in FD population at baseline	After exercise prescription: - The relative maximum performance increased by 12.1%. - The mean of blood lactate at maximum performance increased from 5.4 (78) mmol·L^−1^ to a mean of 7.2 (2.4–10.2) mmol·L^−1^ (*p *= 0.038). - Patients reported increased well-being, daily activity and reduced fatigue
Wang et al. (65)	CPET	In FD women: - Fatigability (58.5%, 24/41) - Exercise intolerance (82.5%, 33/40). - Decrease in Diastolic blood pressure ≥10 mmHg - Reduction of Maximal oxygen consumption - Reduction in maximal heart rate during stress test	N/A
Bierer et al. (66)	CPET	Decrease in diastolic blood pressure of about 10 mmHg.	N/A
Powell et al. (69)	Bruce protocol (treadmill), ramp protocol (cycle ergometer) and CPET.	- Impaired cardiopulmonary exercise capacity measured by CPET. - Lower heart rate at peak exercise, max indexed VO2, and peak index oxygen pulse.	N/A

N/A, not applicable.

### 2.5 Therapeutic strategies for impaired exercise tolerance and fatigue in FD

Enzyme Replacement Therapy has proven to be a life-changing treatment for Fabry disease, significantly improving the prognosis and quality of life for affected individuals. It addresses the underlying enzyme deficiency, slows disease progression, and mitigates symptoms and complications associated with the condition (1, 23, 70, 71). However, the impact of ERT on exercise tolerance in FD patients has been poorly addressed, and the few available data are controversial. Such studies suggest that ERT may positively influence the exercise capacity and cardiopulmonary performance of individuals with Fabry disease, in particular increasing the V0_2_/HR ratio during physical activity (55). Conversely, from other studies, only a modest improvement in the anaerobic threshold emerges for FD patients under ERT, while the V02 max did not change at all (32). The conflicting results could be explained by differences among the study populations (male vs. female) and/or the entity of organ damage, and genetic variants. In a recent report, Tuan et al. assessed the peak exercise capacity of patients with classic vs. cardiac FD variant. The study revealed that patients with cardiac variant experienced a decrease in peak exercise capacity over time, while patients with classic variant-FD showed no significant changes in exercise capacity during the same period. Moreover, ERT appears to have potential benefits in stabilizing exercise capacity in patients with the classic variant and not for subjects with the cardiac variant (72). These findings confirm that the heart plays a critical role in determining exercise capacity in individuals with FD, and that the cardiac variant may have a more profound impact on the exercise tolerance of FD patients and on their response to ERT.

Non-conventional therapeutic strategies should also be employed to manage exercise intolerance and fatigability in FD patients. Exercise tolerance can be trained by exercise prescription. Indeed, exercise therapy is a widely recognized and evidence-based therapeutic approach that utilizes physical activities and exercises to prevent, manage, and rehabilitate various medical conditions. It plays a pivotal role in improving physical function, reducing pain, and enhancing overall well-being for individuals of all ages and fitness levels. It is utilized in various medical settings, including hospitals, clinics, and outpatient facilities, to optimize physical health and improve the quality of life for patients. Exercise therapy also plays a crucial role in managing chronic diseases such as diabetes (73), heart disease (74, 75), and chronic obstructive pulmonary disease (COPD) (76). Regular physical activity has been shown to improve symptoms, control disease progression, and enhance the overall quality of life for patients with these conditions (77). Only one pilot study evaluated the possibility of prescribing exercise in patients with FD. Over one year, the patients underwent an exercise protocol, and 58% of them reported decreased fatigability. Moreover, the study showed that physical performance improved among the patients, with an approximate load increase of 12% (78). This improvement in physical performance suggests that exercise therapy can be employed to enhance functional capacity and overall physical well-being in individuals with FD. However, further studies are needed to support the exercise training of FD patients and also to design a specific and adapted exercise program for this condition. The effects of intervention (ERT or training) on exercise tolerance in FD patients are reported in Table 1.

## 3 Conclusions

Exercise intolerance emerges as a phenotypic hallmark of FD. Even with different extensions, reduced exercise capacity, and fatigue are observed in FD patients with classic and cardiac variants as well, in males and females, in the presence or not of full-blown target organ damage. The exercise capacity is the result of a systemic engagement of different districts including heart, lung, vasculature, and skeletal muscle (Table 2). Likely, the alterations of their synergic work and dynamism occur early, preceding the single target organ abnormalities. Hence, the assessment of exercise capacity could be a precious tool to reveal the early signs of dysfunctions. The current challenge in FD management is the detection of the “silent alerts” which can guarantee a tempestive therapeutic decision, especially in female patients who are undertreated. In this scenario, we propose to employ the evaluation of exercise tolerance by CPET in the routine diagnostic and monitoring process of FD patients, including females. Another important gap that the research should overcome is the study of the effects of available therapies on exercise tolerance, even because fitness capacity is a key aspect of patient quality of life. Moreover, in the current report, we also point out the lack of data on the effects of exercise training in FD population. Hence, we underline the urgent need of research studies specifically focused on potential therapeutic effects of an adapted physical activity program in FD patients.

**Table 2:** Main determinants of exercise intolerance in FD patients.

District	Mechanisms of dysfunction
Cardiac determinants	Cardiac hypertrophy, chronotropic incompetence, functional mitral regurgitation, left atrial dysfunction
Pulmonary involvement	Ventilation abnormalities, obstructive airway disease
Skeletal muscle determinants	Altered metabolic capacity, fibers disarrangement
Neurological determinants	Anhidrosis, neuronal alterations, depression
